# Risk of the high-riding variant of vertebral arteries at C2 is increased over twofold in rheumatoid arthritis: a meta-analysis

**DOI:** 10.1007/s10143-020-01425-w

**Published:** 2020-10-26

**Authors:** Tomasz Klepinowski, Jagoda Cembik, Leszek Sagan

**Affiliations:** 1 Department of Neurosurgery, Pomeranian Medical University Hospital No. 1, Szczecin, Poland; 2 Pomeranian Medical University, Szczecin, Poland

**Keywords:** High-riding vertebral artery, Craniocervical fusion, Atlantoaxial dislocation, Atlantooccipital dislocation, Meta-analysis, Rheumatoid arthritis

## Abstract

Rheumatoid arthritis (RA) might lead to atlantoaxial instability requiring transpedicular or transarticular fusion. High-riding vertebral artery (HRVA) puts patients at risk of injuring the vessel. RA is hypothesized to increase a risk of HRVA. However, to date, no relative risk (RR) has been calculated in order to quantitatively determine a true impact of RA as its risk factor. To the best of our knowledge, this is the first attempt to do so. All major databases were scanned for cohort studies combining words “rheumatoid arthritis” and “high-riding vertebral artery” or synonyms. RA patients were qualified into the exposed group (group A), whereas non-RA subjects into the unexposed group (group B). Risk of bias was explored by means of Newcastle-Ottawa Scale. MOOSE checklist was followed to ensure correct structure. Fixed-effects model (inverse variance) was employed. Four studies with a total of 308 subjects were included in meta-analysis. One hundred twenty-five subjects were in group A; 183 subjects were in group B. Mean age in group A was 62,1 years, whereas in group B 59,9 years. The highest risk of bias regarded “comparability” domain, whereas the lowest pertained to “selection” domain. The mean relative risk of HRVA in group A (RA) as compared with group B (non-RA) was as follows: RR = 2,11 (95% CI 1,47–3,05), *I*^2^ = 15,19%, Cochrane Q = 3,54 with overall estimate significance of *p* < 0,001. Rheumatoid arthritis is associated with over twofold risk of developing HRVA, and therefore, vertebral arteries should be meticulously examined preoperatively before performing craniocervical fusion in every RA patient.

**Electronic supplementary material:**

The online version of this article (10.1007/s10143-020-01425-w) contains supplementary material, which is available to authorized users.

## Introduction

**Fig. 1:**
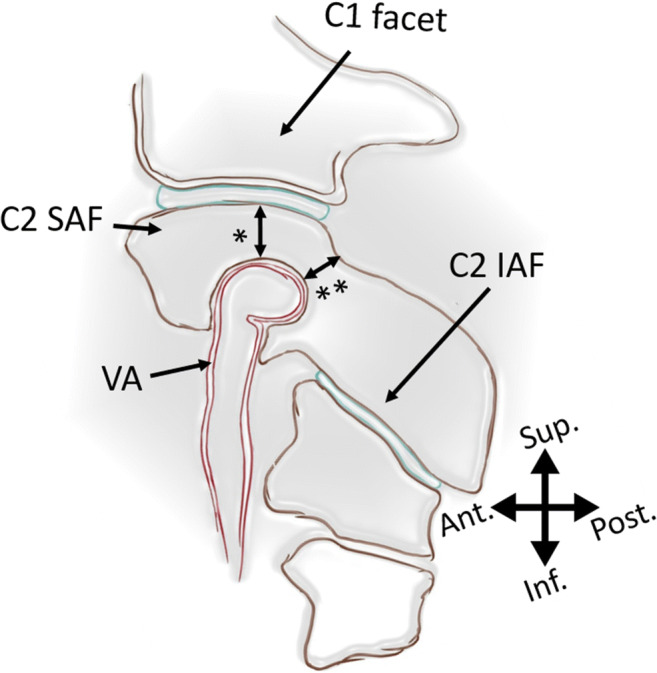
Sagittal section of atlantoaxial facetal joint. High-riding vertebral artery is defined as isthmus height < 5 mm or/and internal C2 height < 2 mm measured at the level 3 mm lateral to the lateral border of spinal canal. SAF, superior articular facet. IAF, inferior articular facet. VA, vertebral artery. *Internal height. **Isthmus height. Ant., anterior. Post., posterior. Sup., superior. Inf., inferior

Rheumatoid arthritis (RA) is an inflammatory autoimmune disease affecting synovial joints as well as other organs with high morbidity and increased mortality [6]. Among many skeletal regions, cervical spine is also often disturbed possibly resulting in atlantoaxial instability [19]. However, little has been written about the immediate surroundings of the vertebral artery at the level of C2 in RA patients (Fig. 1). Mainly, its anomalies are rather congenital than acquired [20]. However, recent data suggests that one of its variants, namely high-riding vertebral artery, might be more common in patients with RA [8]. High-riding vertebral artery (HRVA) is defined as internal height of C2 ≤ 2 mm and/or C2 isthmus height ≤ 5 mm measured at the level 3 mm lateral to the border of spinal canal [16]. Identification of HRVA is crucial before approaching craniocervical junction fusion, as it determines a surgical method [8]. Modality of imaging used to diagnose HRVA is usually computed tomography angiograms (CTA). The overall prevalence of HRVA in general population is 25,3% [8]. It is speculated that in RA it might be higher, approximately 42,9% [8]. The literature, though, is not unanimous, with some papers stating to the contrary [10], and relative risk (RR) has not been estimated yet. Therefore, a timely meta-analysis appears to be contributory. To the best of our knowledge, this is the first attempt in the literature to provide RR of HRVA for patients with RA. The null hypothesis is that the 95% confidence interval RR includes 1, meaning the risk is not increased for RA patients.

## Methods

### Search strategy

Two researchers (neurosurgical resident TK & JC) scanned the following databases: PubMed MEDLINE, Web of Science, EMBASE, SciELO, and China National Knowledge Infrastructure. Search terms included “rheumatoid arthritis” AND “high-riding vertebral artery,” “high-riding vertebral groove,” “axial isthmus,” “high-riding transverse foramen,” “C2 isthmus,” “high-ride vertebral artery,” “HRVA,” “axis isthmus,” and “vertebral artery.” Time span was not restricted. The authors strictly followed MOOSE checklist (Electronic Supplementary Material 1). Titles were imported into Mendeley Desktop 1.19.4, which was also used for creation of the bibliographic data. Next, de-duplication was conducted. References of the included articles were tracked in order to identify any potentially missed papers.

### Eligibility

The following inclusion criteria were imposed: (1) cohort studies with exposed and unexposed groups, (2) patients with rheumatoid arthritis as an exposed group, (3) subjects without rheumatoid arthritis as an unexposed group, (4) information on incidence of HRVA per person, and (5) Neo’s and Bloch’s definition of HRVA (internal height ≤ 2 mm and/or C2 isthmus height ≤ 5 mm measured at the level 3 mm lateral to the border of spinal canal). Of note, the above-mentioned measurements incorporated into the definition of the HRVA had to be taken in sagittal section of computed tomography (either classic or CTA). Exclusion criteria were determined as (1) groups who were operated on by means of unilateral C1-C2 transarticular fixation (in order to avoid a possible bias due to a high risk of unilateral HRVA), (2) papers with overlapping data, (3) case reports, (4) cohort studies with a total number of patients less than 10, (5) letters to the editors, (6) commentaries, (7) conference abstracts, (8) insufficient data, (9) no definition of HRVA, (10) reviews, (11) surveys, and (12) errata. English language was not a condition—articles in other languages were translated by proficient speakers of the given language and then evaluated by the authors. In order to avoid data overlap, studies of the same authors were given a particular attention so as to identify any overlapping recruitment timeline or inclusion criteria.

### Data extraction

Two authors did the data extraction independently into the spreadsheet of Microsoft Excel 2016 (Redmond, USA). Pieces of information aimed to be extracted were as follows: (1) number of subjects without RA, (2) number of subjects with RA, (3) number of non-RA subjects with HRVA, (4) number of RA patients with HRVA, (5) geographical location, (6) age, and (7) sex. If an article was lacking important data, corresponding authors were communicated with.

### Risk of bias assessment

Quality check and risk of bias assessment of the studies included in this meta-analysis were conducted by means of Newcastle-Ottawa Scale (NOS) as well as by evaluation of the funnel plot symmetry. Two researchers (neurosurgical resident TK & JC) assessed each of the included studies separately. In case of discrepancy, a senior neurosurgeon (LS) was called in so as to reach consensus. All articles were evaluated based on three domains: (1) selection, (2) comparability, and (3) outcome. A maximum of stars that could have been awarded for each domain was as follows: for “selection” four stars, for “comparability” two stars, and for “outcome” three stars. Lack of any stars in a given domain meant the risk of bias was high. At least one star but shy of a maximum meant the risk of bias of moderate. A maximum number of stars meant the risk of bias was low. For the sake of “outcome” domain in NOS, an adequate period of the established diagnosis of RA was arbitrarily set to at least 2 years.

### Statistics

Meta-analysis was conducted by means of MetaXL 5.3, EpiGear International Pty Ltd. (Brisbane, Australia) and Statistica 13.3.0, TIBCO Software Inc. (Palo Alto, CA, USA). Relative risk (risk ratio; RR) was calculated with corresponding confidence interval of 95%. Heterogeneity was assessed based on *I*^2^ and chi^2^. *I*^2^ value was interpreted with general accordance: 0 to 40%, not important; 30 to 60%, moderate heterogeneity; 50 to 90%, substantial heterogeneity; and 75 to 100%, considerable heterogeneity. Level of significance for Cochrane Q *p* value was arbitrarily set to < 10% (< 0,10). Level of significance for *p* value of comparative tests was arbitrarily set to < 5% (< 0,05). For heterogeneity lower than 40%, fixed-effects model was planned. In case of heterogeneity ≥ 40%, random-effects model would be adapted.

## Results

### Selection process and study characteristics

**Fig. 2:**
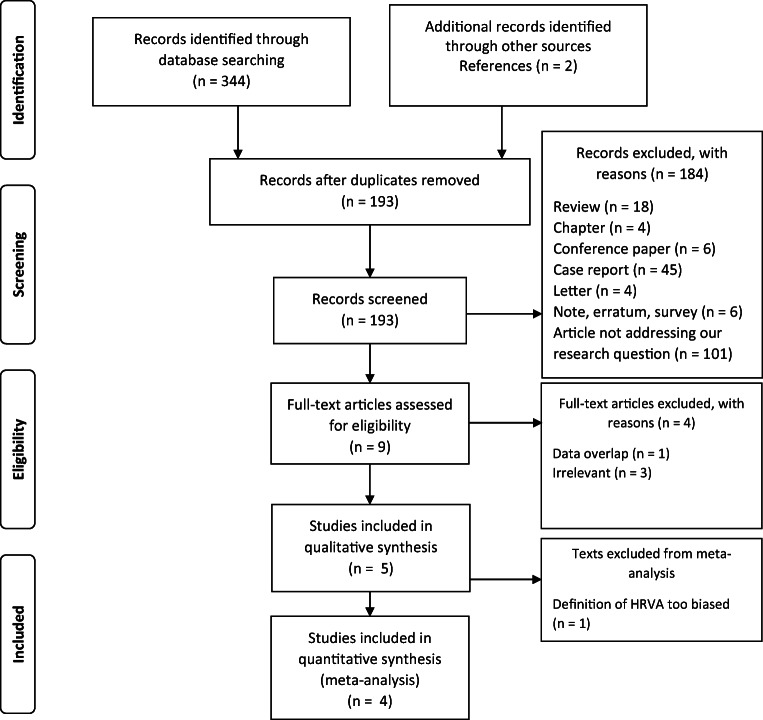
A flow diagram depicting the study selection process

A flow diagram shows steps of the selection process (Fig. 2). Of 346 studies yielded by the initial search (344 from search engines, 2 from relevant references), 193 left once de-duplication process was completed. One hundred eighty-four records are eliminated at the level of title and abstract screening (182 from the search engines, along with 2 articles that were found through references—exclusionary reasons are shown in Fig. 2). Nine full-text articles were assessed for eligibility criteria. Finally, 4 studies were deemed eligible for meta-analysis comprising a total of 308 subjects. One hundred twenty five were in group A (the exposed arm of RA patients), whereas 183 were in group B (the unexposed arm of non-RA subjects) [4, 5, 10, 15]. One study [14] was included in qualitative, but not in quantitative synthesis due to an unacceptable risk of bias; therefore, it is addressed separately in discussion. Mean age in group A was 62,1 years, whereas in group B 59,9 years. All four studies were of Asian origin. Details are presented in Table 1.

**Table 1:** Characteristics of the included studies

Study name	Total cases	RA	RA with HRVA (%)	Non-RA	Non-RA with HRVA (%)
Chung 2005	67	16	10 (62,5%)	51	17 (33,3%)
Higashino 2009	129	90	31 (34,4%)	39	3 (7,7%)
Lee J 2007	12	3	0 (0%)	9	3 (33,3%)
Moon 2018	100	16	9 (56,3%)	84	22 (26,2%)

*RA* patients with rheumatoid arthritis. *HRVA* high-riding vertebral artery

### Risk of bias and quality assessment

**Fig. 3:**
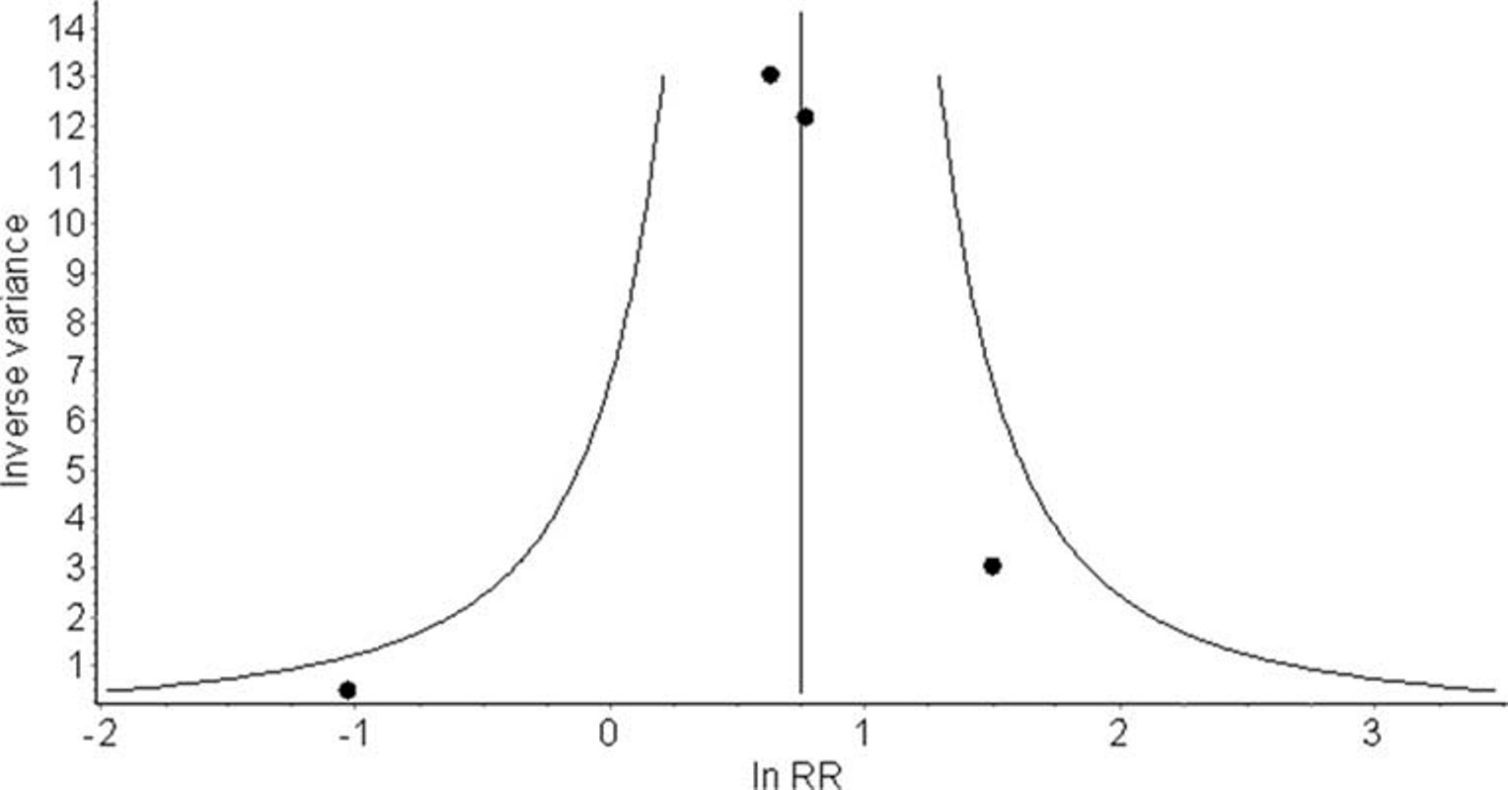
A funnel plot illustrating quantitative assessment of the risk of bias

Summary of the Newcastle-Ottawa Scale for each study included in the meta-analysis is shown in Table 2. In “selection” and “outcome” domains, all studies presented a moderate risk of bias. On the other hand, within the “comparability” domain, most of the studies presented a high risk of bias. Demonstration that outcome of interest (HRVA) was not present at the beginning of the studies was not feasible as it would have required CT scans prior to establishing diagnosis of RA. The funnel plot (Fig. 3) indicates that quantitatively only one study [10] strongly deviated from the rest, yet it had the least impact upon the mean RR.

**Table 2:** Tabular display of the Newcastle-Ottawa Scale summarizing qualitative evaluation of the risk of bias

Study name	Selection	Comparability	Outcome
Chung 2005	**✵✵**		**✵✵**
Higashino 2009	**✵✵✵**	**✵**	**✵✵**
Lee J 2007	**✵**		**✵**
Moon 2018	**✵✵**		**✵**

A maximum number of stars that could be awarded was as follows: four for selection, two for comparability, three for outcome

### Estimated pooled effect

**Fig. 4:**
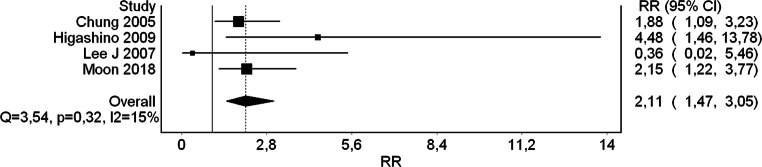
A forest plot of the analyzed studies indicating that rheumatoid arthritis is a risk factor for high-riding vertebral artery

The combined relative risk estimate was RR = 2,11 (95% CI 1,47–3,05), *I*^2^ = 15,19%, Cochrane Q = 3,54, *p* = 0,32. Test for overall effect was significant with *p* < 0,001. The forest plot indicates mean distribution of RR across the studies along with the pooled effect (Fig. 4).

## Discussion

Although several papers indicated that RA might pose a risk of developing HRVA, some small studies stated to the contrary. In a small study of Lee J et al., none of three RA patients had HRVA [10]. Their study had little impact upon the overall pooled estimate, though. Studies of larger samples and lower risk of bias state that HRVA are clearly more common in RA patients. This meta-analysis confirms that, according to the current data, the relative risk is increased approximately twofold.

### Presumed pathophysiology explaining the findings

Findings of this meta-analysis might be elucidated by progressive reduction of volumetric bone mineral density [18]. RA is known to induce osteoporosis in multiple ways [11]. It might be either systemic, periarticular, or focal. Thinning of the isthmus appears to combine all these three types of osteoporosis. Periarticularly, there is significant loss of trabeculae in number and in size stemming from prolonged secretion of pro-inflammatory cytokines, such as tumor-necrosis factor and interleukin 6. Systemically, disturbed osteoblastogenesis and enhanced osteoclastogenesis lead to generalized resorption of both cortical and trabecular bone. Locally, weak and demineralized bone of C2 vertebra, with transverse foramen located close to the superior articular facet, is being constantly thrusted into by the pulsating VA, over time creating a local effect of furrowing. This erodes the isthmus, rendering it insufficient in terms of screw placement. Evident cervical erosions in conventional radiographs are noticed in 16% of RA patients at 9-year follow-up [2] but much more often in CT scans [8].

### Craniocervical fusion in RA patients

Rheumatoid patients’ atlantoaxial joints are prone to inflammation, subluxation, and dislocation [13, 21]. Formation of pannus around the dens of C2 as well as dens invagination into the foramen magnum might lead to craniocervical stenosis and subsequently to myelopathy of the bulbospinal junction. Many approaches are available for dens pathologies in rheumatoid arthritis including transoral release or extreme lateral [1]. Currently, treatment shifts from transoral release and posterior fusion toward solely posterior fusion, as it has been proven that craniocervical fixation leads to pannus resolution at follow-up visits [9]. As craniocervical dislocation in RA patients is often precipitated by trauma, its management shares some similarities with post-traumatic patients: for both, levels of fusion are most often C1-C2, followed by occiput-C2 [3, 7]. Precise insertion of the screws is of a paramount importance. Failure to notice a high-riding vertebral artery preoperatively at the planning stage may lead to choosing a risky method of craniocervical fusion, ultimately ending up in injuring the VA, massive blood loss, and neurological deficits. Recently, a detailed guide for clinical decision-making was provided, which facilitates choice of fusion method depending on the side of dominance of VA and whether HRVA is unilateral or bilateral [8]. In ipsilateral HRVA and dominant VA, it is recommended to either avoid transarticular/transpedicular screw or proceed only with spinal navigation.

### Limitations

A small number of studies are a limitation of the present meta-analysis. One potentially contributory cohort study of Miyata et al. [14] had to be rejected from quantitative meta-analysis at the level of eligibility evaluation due to imprecise definition of HRVA, as it would have possibly introduced an unacceptable risk of bias (“the maximum screw diameter that could be inserted through the isthmus of the axis without breaching the cortex”). This peculiar definition was followed by oddly high incidence of HRVA (70,2% in RA vs 15% in non-RA); therefore, the authors of the present meta-analysis decided not to include it quantitatively but only consider it in qualitative pondering. Regional differences in HRVA definition are prominent. For instance, Paramore et al. [17] from North America named vertebral arteries high-riding if the transverse foramen was on the path of ideal transarticular screw trajectory. As useful as it might appear in day-to-day clinical practice, it is not optimal for comparative research studies due to lack of standardization. European studies are also not unanimous. Meyer et al. [12] listed 7 patients with HRVA but did not provide any definition of it, whereas Czech study of Vaněk et al. [20] adhered to the Asian definition (internal height ≤ 2 mm and/or C2 isthmus height ≤ 5 mm measured at the level 3 mm lateral to the border of spinal canal). Besides, among other limitations, all four studies coming from Asia may question validity of the results in other populations. Therefore, more studies in the future are necessary exploring relationship between rheumatoid arthritis and high-riding vertebral artery in the remaining continents. Acknowledging this issue, the authors of this meta-analysis have commenced a CT-based cohort study on HRVA of the East European population. Moreover, it is worth mentioning that 2 of the studies had relatively wide range of 95% CI, putting significant weight of the pooled RR into the other 2 studies with narrow 95% CI (Fig. 4). This weakens the findings of the meta-analysis highlighting the need for more studies. Additionally, as shown in the tabular display of NOS (Table 2), the currently available data, although the best we have now, is flawed with methodological bias, especially in terms of comparability.

## Conclusion

Rheumatoid arthritis is associated with an increased risk (RR = 2,11 [95% CI 1,47–3,05]) of developing a high-riding vertebral artery. Therefore, VAs should be thoroughly examined on CT angiograms before performing craniocervical fusion in these patients. The presence of HRVA might influence choice of approach. More research studies with better methodology encompassing subjects outside Asia are needed in order to extrapolate the results.

## Electronic supplementary material

ESM 1 (DOC 71 kb)
